# Increasing stress resilience in older adults through a 6-week prevention program: effects on coping strategies, anxiety symptoms, and cortisol levels

**DOI:** 10.3389/fpsyg.2024.1499609

**Published:** 2025-01-06

**Authors:** Marie-Josée Richer, Sébastien Grenier, Sonia Lupien, Pierrich Plusquellec

**Affiliations:** ^1^Psychoeducation School, University of Montreal, Montreal, QC, Canada; ^2^Laboratoire d’étude sur l’anxiété et la dépression gériatrique, Centre de recherche de l’institut universitaire de gériatrie de Montréal, Montreal, QC, Canada; ^3^Psychology department, University of Montreal, Montreal, QC, Canada; ^4^Center for Studies on Human Stress, Centre de recherche de l’institut universitaire en santé mentale de Montréal, Montreal, QC, Canada; ^5^Psychiatric Department, University of Montreal, Montreal, QC, Canada; ^6^Centre d’études en sciences de la communication non verbale, Centre de recherche de l’institut universitaire en santé mentale de Montréal, Montreal, QC, Canada

**Keywords:** older adults, stress, cortisol, psychological distress, stress management intervention, coping strategies, anxiety

## Abstract

**Introduction:**

As people age, chronic stress, resulting in prolonged or repeated activation of the hypothalamic–pituitary–adrenal (HPA) axis, has been associated with long-term adverse health outcomes. Coping strategies and social support have been recognized as contributing to resilience to stress in older adults. Few studies have evaluated stress management training (SMT) interventions based on psychoneuroendocrinology that were designed to be delivered to healthy older adults in community settings.

**Methods:**

In this study, a total of 170 older adults (mean age = 76.07, SD = 7.67) participated in a cluster-randomized trial designed to compare the delivery of an SMT intervention with a waitlist condition.

**Results:**

The effect of SMT on coping strategies, stress, anxiety, and depression was measured 3 weeks and 3 months after the intervention. In addition, we tested the effect on basal cortisol secretion over 2 days from saliva samples upon awakening and the total diurnal cortisol output [area under the curve with respect to ground (AUCg)]. Results from repeated measures analyses of variance showed that participants who received the intervention demonstrated a significant increase in problem-solving coping strategies and a decrease in anxiety scores 3 weeks after the intervention compared to the waitlist group. STM participants also demonstrated lower cortisol levels on the AUCg index. At the 3-month follow-up, gains were maintained only on the AUCg index.

**Discussion:**

This type of brief preventive program could reduce basal cortisol levels in older adults, which may be an important protective factor against health outcomes associated with chronic HPA activation. Our results provide sufficient evidence to warrant further research to improve the effectiveness of *O’stress* in different settings.

## 1 Introduction

The World Health Organization (2020) defines healthy aging as “the process of developing and maintaining the functional capacity that enables well-being in older age” (p. 8). To promote healthy aging, researchers have explored the function of numerous determinants of health and well-being in old age (for a review, see Cardinal et al., 2008). Stress influences health, disease, and well-being and thus has an important place in models of healthy aging. Research to improve the management of stress responses in old age has been identified as a key priority in the field (Kennedy et al., 2014; Polsky et al., 2022).

*Resilience to stress* has been described as the ability to adapt and maintain one’s psychological health in the face of adversity across the lifespan (Troy et al., 2023). More recently, researchers have acknowledged the complex nature of psychological resilience, thus highlighting the multifaceted processes that it encompasses (Vella and Pai, 2019). In a process-based framework, psychological resilience involves personal competencies and characteristics, and the context and time in which they are displayed (Troy et al., 2023). The study of aging places particular emphasis on understanding resilience to stress, as individuals may undergo significant changes in physiological, psychological, and social roles during late adulthood (American Psychological Association, 2017). The scientific literature reports several types of stressors at this stage of life. In a sample of 282 adults aged 54 to 91 years (M = 68.79, SD = 5.10), Scott et al. (2011) identified the combined contribution of life events, neighborhood strain, age-related discrimination (i.e., ageism), social isolation (i.e., loneliness), financial strain, and physical health status to the experience of perceived stress. In a large study involving two samples of over 6,000 Americans aged 50 and over (M = 67 years old), the authors examined the impact of different types of stressors on the cognition. The most common sources of chronic stress included health problems (9.3 to 10.3%), physical or emotional problems in spouse or child (7.9 and 7.4%), and financial strain (4.9, 6.7%; Wang et al., 2022). A more recent study of 127 adults aged 60 to 95 years (M = 79.4, SD = 9.15) highlights the subjective aspect of stressors during aging through the contribution of individuals’ perceptions of their health and aging. Indeed, individuals who had more negative global and daily perceptions of their health and aging experienced greater perceived stress (Whitehead and Blaxton, 2021). As demonstrated by Wettstein et al. (2021), the relationship between perceptions of aging and stress is a reciprocal one, as stress also affects perceptions of aging.

*Stress* is a biological response that occurs when one’s external or internal balance (homeostasis) is challenged. The main hormonal circuit involved is the hypothalamic–pituitary–adrenal (HPA) axis. The end product of HPA activation is cortisol (Selye, 1936). In fact, cortisol is continuously secreted in the body according to a circadian rhythm (Brown and Wilkinson, 2015). Animal and human studies have shown a progressive increase in circadian cortisol concentrations in the first part of the night, with circulating cortisol levels increasing with age (Duffy et al., 2015). In a review, Goncharova (2020) mentioned that hyperactivation of the HPA axis has been found in older adults and in aging primate models. Another trend in aging research reported in two reviews (including studies in humans, nonhuman primates, and rodents) is a decrease in the strength of negative feedback from the glucocorticoids, such that the aging body is overexposed to cortisol while the process of regaining homeostasis is slowed, suggesting a limited psychophysiological coping capacity (Goncharova, 2020; Rao and Androulakis, 2019). Goncharova (2020) and Rao and Androulakis (2019) also report that age-related changes in the circadian rhythm of cortisol have been shown to also affect HPA reactivity, i.e., the cortisol response to stress exposure (e.g., by increasing HPA reactivity or decreasing the ability of the stress system to quickly return to a resting level).

Stressors come in both explicit and implicit forms. Explicit stressors include situations that almost everyone perceives as threatening. Relative threats, as opposed to absolute ones (i.e., those arising from life-threatening events), arise from a sense of novelty, its unpredictability, a threat to one’s ego, and a sense of lack of control over a situation (N.U.T.S.; Dickerson and Kemeny, 2004; Mason, 1968). They depend on the individual’s interpretation of the situation. These relative stressors can be acute (e.g., being late for an appointment) or more insidious, such as daily annoyances (e.g., frequent arguments with a partner) or chronic stress (e.g., caregiving; Epel et al., 2018). Individuals may also experience implicit stressors in interpersonal situations that trigger an automatic physiological stress response. Stress contagion is rooted in the phenomenon of emotional contagion (Hatfield et al., 2014), which can be defined as “the tendency to automatically mimic and synchronize facial expressions, vocalizations, postures, and movements with those of another person and, consequently, to converge emotionally” (Hatfield et al., 1994, p. 153). Physiological resonance is one of the primary mechanisms involved. Buchanan et al. (2012) demonstrated the contagion of the physiological response to acute stress between a target and an observer behind one-way glass. In their study, observers showed an increase in their cortisol (stress hormone) response that was proportional to that of the individuals undergoing social stress, illustrating the resonance of the stress response at the physiological level. When the observer and the target were significant others, Engert et al. (2014) and Engert et al. (2018) found a similar and even stronger resonance. We posit that in aging, individuals may find themselves in contexts that could triggers stress contagion such as caregiving and living in residences for retirement.

In short, age itself has an impact on the functioning of the stress system, but another important element is the burden of chronic stress that some older people have experienced throughout their lives, which may have worn down their stress system. In contrast to the stress response to acute stressors, the presence of prolonged or repeated stressors results in prolonged or repeated activation of the HPA axis, which may have long-term deleterious effects (Juster et al., 2011; Turner et al., 2020). Many studies have examined the effects of chronic stress in older adults and have identified an extensive list of health concerns that are particularly vulnerable to chronic stress.

The physiological changes that occur in the face of chronic stress can promote some diseases such as hypertension, type 2 diabetes (T2D), and cardiovascular disease (Kelly and Ismail, 2015; Osborne et al., 2020; Seiler et al., 2020; Steptoe and Kivimaki, 2012). Dysfunction of the hypothalamic–pituitary–adrenal (HPA) axis leads to increased secretion of glucocorticoids. This, in turn, disrupts glucose metabolism by impairing hepatic glucose uptake and insulin secretion, thereby contributing to insulin resistance and promoting inflammation, among other consequences. This pathway increases the risk of hypertension and T2D, which are also known risks for cardiovascular disease (Osborne et al., 2020; Sharma and Singh, 2020). Several combined mechanisms are also at play: the stress response includes increased sympathetic nervous system (SNS) activity and an opposite response in parasympathetic nervous system (PSNS) activity; increased leukopoiesis (i.e., the accumulation of fats, cholesterol, and other substances in and on the walls of the arteries); altered vascular reactivity and increased coagulation, and immune dysregulation leading to inflammation and to immunosuppression. All of these responses to chronic stress could make the body more susceptible to inflammatory diseases such as cardiovascular disease (Osborne et al., 2020). Moreover, chronic stress has been recognized for its impact on cognitive function. Although findings have varied over the years due to differences in the methods used to assess stress, evidence suggests that changes in the brain—particularly the hippocampus—resulting from chronic activation of the HPA axis may be associated with an increased risk of cognitive decline (Franks et al., 2020). A systematic review and meta-analysis of 26 and 16 studies conducted between 2003 and 2020, respectively, examined the association between adult stress-including life events and perceived stress-and the risk of cognitive impairment and all-cause dementia (Franks et al., 2020). Across studies, findings from Franks et al. (2020) and Luo et al. (2023) suggest that both stressful life events and perceived stress are associated with cognitive decline and an increased risk of all-cause dementia. Finally, stress is associated with a higher risk of reporting depressive and anxiety symptoms (Duric et al., 2016; Grolli et al., 2021; Mantella et al., 2008).

In contrast, research suggests that psychological resilience may be protective for older adults in the face of adversity. A large longitudinal study, involving two samples of 11,050 (mean age = 67.53, SD = 9.21) and 12,823 (mean age = 67.53, SD = 9.58) individuals aged 51 to 104 years, demonstrated that psychological resilience exerts independent and significant protective effects on health transitions and trajectories (Taylor and Carr, 2021). These effects were observed across five different health outcomes, including perceived health, functional limitations, activities of daily living, and depressive symptoms (Taylor and Carr, 2021). Despite inconsistent findings, other studies suggest that psychological resilience supports the preservation of cognitive functions during aging. For example, one study found that individuals (mean age of 55 years) who were rated as resilient had higher cognitive function 5 years later. Notably, this association was significant only among men with lower levels of education and was independent of age (Yang et al., 2021).

With increasing longevity, older adults are bound to face more challenges, underscoring the need for enhanced stress resilience among them. This highlights the importance of implementing stress management interventions in practice. The majority of stress management programs evaluated for the general population have included cognitive-behavioral stress management (CBSM; Hammerfald et al., 2006; Sharifirad et al., 2013; Tang et al., 2020) and mindfulness-based interventions (Khoury et al., 2015; Querstret et al., 2020). CBSM programs are usually based on the appraisal model of Lazarus and Folkman (1984). As for mindfulness methods, they are rooted in the practice of meditative techniques (Querstret et al., 2020). Both types of interventions frequently incorporate strategies recognized for their impact on altering the perception of stress or modulating the functioning of the HPA axis. These strategies include recognizing stressors and their effects on the mind and the body, breathing exercises, promoting problem-solving and emotion-focused coping behaviors, and cultivating the ability to identify internal and external coping resources, such as those within one’s social network. Meta-analyses of stress management interventions in different populations suggest that some interventions have been moderately successful in reducing perceived stress (Chiesa and Serretti, 2009; Querstret et al., 2020; Richardson and Rothstein, 2008). Unfortunately, few studies have evaluated the effectiveness of stress management interventions specifically in older adults. For example, a meta-analysis of 23 studies (including six randomized controlled trials) found a mindfulness-based stress reduction program to be more effective than the control group in reducing depressive symptoms in older adults immediately after the intervention, but their results provided no evidence of reduced anxiety and perceived stress. In addition, the effects on depressive symptoms dissipated at follow-up (Li and Bressington, 2019).

It’s worth noting that few programs are aligned with the past 35 years’ discoveries in the field of psychoneuroendocrinology. This field of research is concerned with understanding the causes and consequences of physiological reactivity to psychological stress in humans, such as the relative stressors (i.e., N.U.T.S.) and their effects on the body. Similar to the mindfulness approach, the Brief Training of Psychoneuroendocrinoimmunology-Based Meditation (BTP-BM) emphasizes the need for education about the stress system and its connections to emotions, cognition, and their biological aspects, while encouraging observation of their effects on behavior (Bottaccioli et al., 2014). The Center for Studies on Human Stress also developed programs rooted in the findings of psychoneuroendocrinology, combined with cognitive-behavioral strategies such as problem-solving and ways to use one’s social network as a coping strategy. These programs have demonstrated reductions in participants’ stress (both perceived and physiological), as well as in participants’ symptoms of distress among youth and social workers (Fecteau et al., 2018; Lupien et al., 2013; Massé and Plusquellec, 2018; Plusquellec et al., 2016).

The scientific literature that focuses on factors that moderate the effects of stress in older adults reports that they are less likely to experience distress symptoms when they use problem-solving strategies, social support–seeking coping styles, and positive reappraisal or cognitive reframing (Cosco et al., 2017; Kraaij et al., 2002). For example, Park and An (2016) examined the moderating effect of coping strategies on the relationship between stress (described as having a “loss experience”) and symptoms of depression in a sample of 156 women and 116 men over the age of 60. They found that problem-focused coping styles moderated the relationship between loss and depression in men.

To our knowledge, no stress management training for older adults in community settings has combined psychoneuroendocrinology findings with a cognitive-behavioral approach. The present study tested the efficacy of an adaptation of the program developed by the Center for Studies on Human Stress in a sample of older adults.

## 2 Objective

This study aimed to evaluate the effects of a stress management training (SMT) intervention in a sample of community-dwelling older adults on problem-focused coping strategies, mental health indicators (e.g., reductions in self-reported stress, anxiety, and depressive symptoms), and physiological indicators of stress (i.e., cortisol levels). Comparisons between the effects of SMT and a waitlist condition were made 3 weeks after the intervention and at a 3-month follow-up.

## 3 Method

### 3.1 Procedure

A cluster randomized trial (CRT) design was used to compare the delivery of the SMT intervention with a waitlist condition. In the context of this study, a CRT was preferred because of the significant potential for contamination in the study, as participants either lived in the same residence or attended the same programs at community organizations (COs). The residences or programs at the COs were randomly assigned to conditions prior to recruitment by a team member not involved in the study. Both the SMT and the waitlist control groups were followed for 3 months, with data collected through repeated questionnaires and saliva samples (at baseline, 3 weeks post-intervention, and at a 3-month follow-up). Participants in the control group were offered the full intervention after completing questionnaires at all time points.

### 3.2 Participants

**Figure 1:**
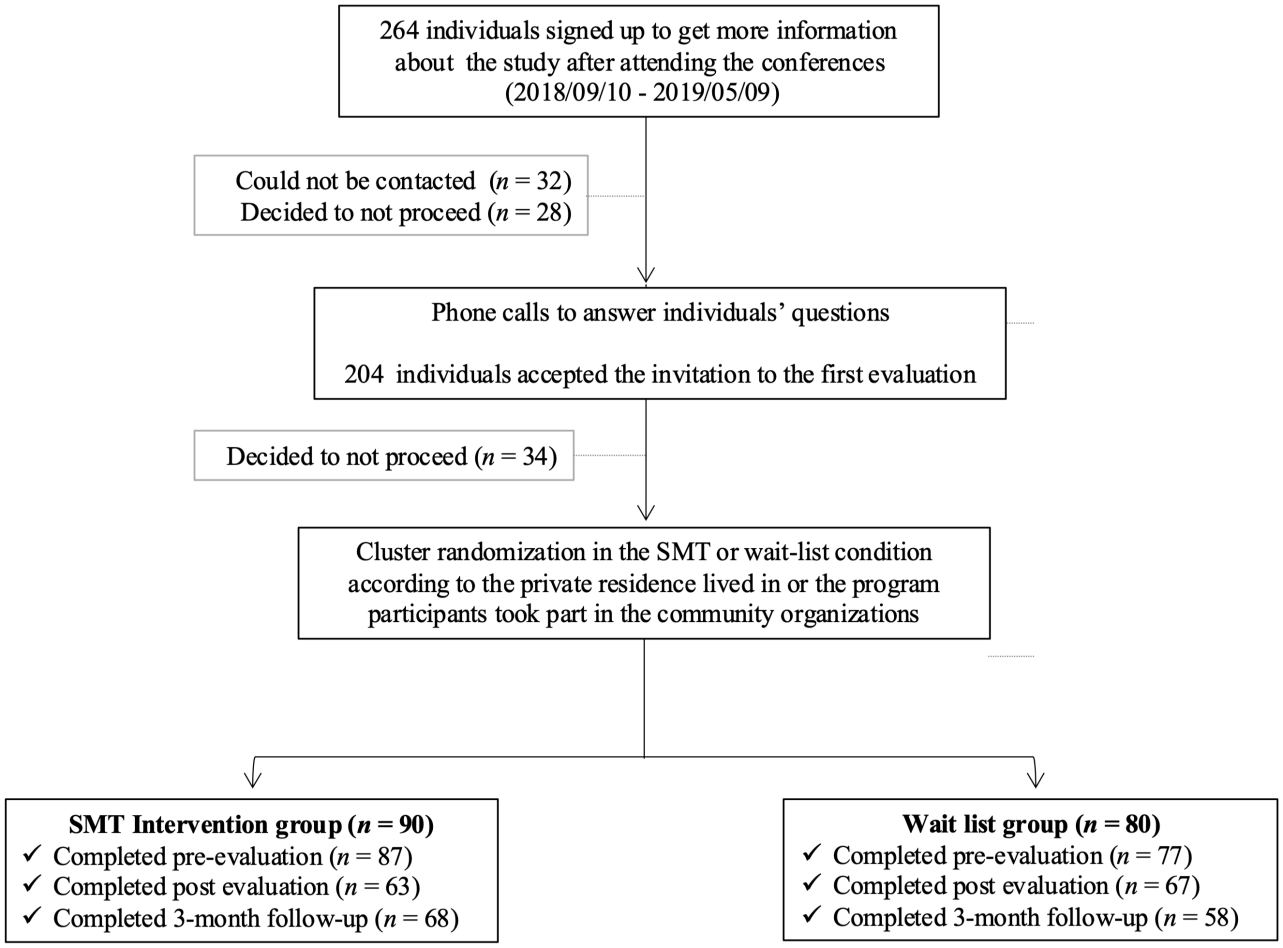
Recruitment process flowchart.

Participants were recruited at private residences (PRs) for retired people with services (health support, security, domestic help, and leisure activities), at a CO offering programs related to caregiving, and at a CO offering a wide range of programs for older adults in the community (support groups for caregivers and people with chronic illnesses or in need of stimulation, residential facilities for retired people, etc.). Randomization was as follows: the experimental condition included participants from four residences and five programs from both COs; the waitlist condition included participants from five residences and three comparable programs from the same two COs. To participate, applicants had to meet the following five inclusion criteria: (1) use services from an organization serving an aging population, (2) be 55 years of age or older, (3) understand spoken and written French, (4) want to better manage stress, and (5) be available to participate in six stress management workshops and three evaluation sessions. Participants enrolled in the study after attending conferences called “Stress and Aging.” The conferences (*n* = 16) were held at PRs and COs and were given by the study’s first author (M.-J. R., a Ph.D. student). During recruitment, which took place between September 2018 and May 2019, 264 people registered to receive more information about the study. Details on the flow of applicants and participants through the recruitment process are provided in Figure 1. Applicants were contacted by phone to review the study and were given time slots in their schedules for assessment sessions and a group formation workshop. Two hundred and four individuals agreed to participate in small group sessions of 5 to 10 people were written consent was obtained and the initial assessment was explained and completed. Thirty-four decided not to participate in the study at the meeting for a variety of reasons (e.g., hospitalization or health issues, stressful life events, difficulty completing the questionnaire even with the assistance of a research assistant, scheduling conflicts). A total of 170 participants consented to the study and completed the sociodemographic questionnaire. The study was approved by the Human Research Ethics Committee of the University of Montreal (Québec, Canada; No. CERAS-2017-18-018-P).

### 3.3 Description of the intervention

The goal of the program being evaluated is to prevent the harmful effects of explicit and implicit stress on participants. The program, developed by CESH, aims to: (1) provide knowledge about stress, its biological process (hormones and physiological reactions, effects on the body), (2) identify stress triggers, (3) recognize physiological reactions in stressful situations, (4) teach emotion-focused strategies for managing stress, and (5) teach problem-solving strategies for coping with stress. The program also addresses the impact of social relationships on stress management. Specific to stress contagion, participants in the intervention learned (1) the role of emotional contagion in their relationships with others, (2) how to recognize the signs of stress contagion, (3) how to identify high-risk contexts (e.g., helping a suffering loved one) and protective contexts (e.g., a loved one whose happiness is contagious), and (4) how to apply the coping strategies learned in the context of stress contagion.

The theoretical underpinning of the program’s activities come from the field of psychoneuroendocrinology and the cognitive-behavioral approach. The intervention includes an educational section that teaches basic concepts about stress, including the physiological process, the hormones involved, their roles, and their effects on the body. Participants are introduced to the science of stress to demystify what happens in their bodies when they are stressed. The program then introduces the factors that lead to a stress response, such as the perception of novelty, unpredictability, threat to one’s ego, and a sense of lack of control over a situation represented by the acronym N.U.T.S. (Lupien et al., 2013; Plusquellec et al., 2016). Finally, the program includes the practice of various stress management strategies. Some strategies target the physiological manifestations of stress, such as abdominal breathing (using various techniques) and physical exertion, while others address stress factors using a problem-solving model (including cognitive assessment of stressors based on the N.U.T.S. factors).

In short, participants are exposed to the mechanisms that describe the biological process of stress and the coping strategies for dealing with a stressor, rather than just solutions. This approach allows for (1) the normalization of the stress response; (2) the deconstruction of myths associated with it; (3) the adoption of a positive or neutral attitude toward stress manifestations, which are no longer seen as symptoms of illness; and (4) the acquisition of a variety of meaningful strategies that participants can select according to their needs in any given context.

The original group workshops of the *DeStress for Success©*program were established in 2007 by the CESH. They were initially designed for adolescents transitioning from primary to secondary school. The program originally consisted of five 50-min workshops, delivered interactively by a facilitator to groups of youth once a week. The program includes a workbook to help generalize the concepts covered. More than three thousand certified facilitators across Quebec have since been trained by CESH to deliver the program throughout the province and throughout the French-speaking world. The mental health benefits observed in youth following their participation in *DeStress for Success©* qualified the program for inclusion in the Canadian Best Practices Portal. Adaptations were evaluated for youth receiving services at the Centre jeunesse de Montréal—Institut universitaire (Plusquellec et al., 2016) and for youth with autism spectrum disorder (Fecteau et al., 2018).

In the context of interventions with older people, the group modality of the program had the advantage of being short in duration and easy to implement. That is, facilitation could be done by a clinician or even a peer helper. Additionally, the group format allowed for the creation of social connections, which is a protective factor for stress management in older adults (Belanger et al., 2016; Kelly et al., 2017). As described in more detail below, the program was adapted to meet the specific needs of the older adult clientele. To distinguish the adapted program from its original version, it was renamed *O’stress* for the purposes of this study.

The adaptation for an older clientele was carried out based on recommendations from the scientific literature and the clinical experience of the research team members to enhance the participant experience (Stanley et al., 2003; Wetherell et al., 2003; Wetherell and Unutzer, 2003). The main adaptations of the program for an older clientele included (1) adding the effects of stress on an aging body and brain; (2) adjusting the vocabulary and examples; (3) modifying some of the interactive activities; (4) adding a sixth workshop on implicit stressors, specifically stress contagion; (5) lengthening the sessions from 50 min to 60–75 min to allow for a sharing period; (6) limiting the group composition to 5–8 participants; and (7) giving participants a notebook with exercises and key concepts to facilitate the learning process. The adapted program was renamed *O’stress*. From a didactic standpoint, the following tools were developed and/or adapted: (1) a facilitator’s guide, (2) six presentation slideshows (one per workshop), and (3) a participant’s workbook. Its contents are described in Table 1.

**Table 1:** Summary of the *O’Stress* program content as adapted from the DeStress for Success© program.

Session number and title	Topics covered
1: Recognizing stress: N.U.T.S.	What is stress? Elements of stress The N.U.T.S. model of stress Test: My sensitivity to N.U.T.S.
2: Application of the N.U.T.S. stress model	Application of the N.U.T.S. model to identify and deal with daily stressors Individual interpretation of stressful events
3: The body’s response to stress	Recognition of the body’s responses to stress and their function Energy mobilization Ways the body gets rid of built-up energy Strategies to cope with stress (emotion-focused coping)
4: Dealing with stress	Using the N.U.T.S. model to deconstruct real-life stressors Coping strategies (problem-focused coping)
5: The importance of others: consolidating our social network	Demonstration of social support versus social pressure as a stress-coping strategy Analyze the social network to identify contexts/individuals where support and pressure are felt when stressed. Reconstruct the resources from the network based on that analysis.
6: Stress contagion	What is stress contagion? Identifying contexts in which contagion of stress appears Applying the coping strategies learned in the context of stress contagion

N.U.T.S. = Novelty, Unpredictability, Threat to ego, Sense of low control.

### 3.4 Measures

Self-report questionnaires were used to collect sociodemographic information and to measure psychological indicators during group sessions facilitated by the study’s first author (M-.J.R.). In addition to the group sessions, several modalities were offered to the participants according to their needs: one-on-one support (for individuals with visual impairment or coordination difficulties, e.g., writing), extra time, and the possibility to complete the questionnaire at home to improve concentration, with easy access to M.-J. R. by phone if needed.

#### 3.4.1 Outcome measures

##### 3.4.1.1 Problem-solving coping strategies

To measure changes in problem-solving coping style in the presence and anticipation of stress, we used the Proactive Coping Inventory (PCI; Greenglass et al., 1999). The PCI measures seven dimensions of coping. In this study, two dimensions—proactive coping and strategic planning—were chosen to assess changes in problem-solving skills because this is a key component of the intervention. Proactive coping (13 items) involves autonomous goal-setting with self-regulatory goal attainment cognitions and behaviors (e.g., “When I experience a problem, I take the initiative to solve it”). One item was removed from the original 14-item scale because it was related to the workplace, which did not apply to our participants. Strategic planning (4 items) measures the tendency to create a goal-oriented plan of action by breaking large tasks into manageable components (e.g., “I break down a problem into smaller parts and do one part at a time”). Respondents must rate the items on a scale ranging from 1 (*not at all true*) to 4 (*completely true*). The scales represent the items’ mean score, from 1 to 4. A high score represents a person’s strong belief in the potential to change a situation that will lead to an improvement of one’s situation/environment. This instrument was translated into French by members of our research team using the forward/backward translation method. The reliabilities (Cronbach’s αs) of the proactive coping and strategic planning scales had adequate values of 0.69 and 0.66, respectively, and were consistent with studies conducted by the authors of the instrument (Greenglass et al., 1999; Pasikowski et al., 2002).

##### 3.4.1.2 Perceived stress

Perceived stress was measured using the Perceived Stress Scale (PSS; Cohen et al., 1983), which was validated by Ezzati et al. (2014) with older adults over the age of 70. The PSS was designed to measure “the degree to which individuals rate situations in their lives as stressful (p. 385)” (Cohen et al., 1983). Validation with older adults showed good psychometric properties for a version with 13 items instead of 14 (Cronbach’s α = 0.83). The item 12 “How often have you been thinking about things that you have to accomplish?” was removed based on the factorial analyse suggesting that older adults interpreted that statement both as a negative and positive take on stress. In addition, the questionnaire showed good sensitivity for detecting perceived stress in participants without cognitive impairment and with mild cognitive impairment (Ezzati et al., 2014).

##### 3.4.1.3 Anxiety and depression

The Hospital Anxiety and Depression Scale (HADS) is a self-report instrument that was developed to assess symptoms of anxiety and depression in nonpsychiatric patients (Zigmond and Snaith, 1983). The Anxiety and Depression subscales each contain 7 items. The items are scored on a four-alternative response scale ranging from 0 to 3, with 0 representing the absence of symptoms. For example, for the statement, “I look forward to things with enjoyment,” the respondent can answer “0—As much as I ever did,” “1—Rather less than I used to,” “2—Definitely less than I used to,” or “3—Hardly at all.” Responses are summed after reversing the scores of six questions. We preferred this instrument to the scales developed specifically for older adults for two main reasons: (1) it is widely used in different settings, including screening of older adults in the community, which provides an advantage for comparison purposes (Djukanovic et al., 2017; Gale et al., 2010), and (2) it assesses the psychological aspects of somatic symptoms of depression to reduce the influence of physical illness (Zigmond and Snaith, 1983). We used the French version of the questionnaire, which has been validated in a large primary care population, including one aged 65 years and older (Roberge et al., 2013). The reliabilities (Cronbach’s αs) of the Anxiety and Depression scales in our sample were 0.82 and 0.72, respectively.

##### 3.4.1.4 Diurnal salivary cortisol

Participants were provided with saliva collection tubes (Sarstedt Tubes, Part No. 62.558.201) and given detailed verbal and written instructions. A video illustrating the procedure was also shown to participants prior to the first collection. Participants were instructed not to smoke on the day of the collection and not to eat, drink, or brush their teeth for 1 h prior to saliva collection. In addition, participants were instructed to provide saliva samples on two nonconsecutive days at awakening, 30 min after awakening, at 4:00 pm, and at bedtime. Participants were given a diary to record the exact time of each collection. All participants that returned a completed saliva kit (at least four samples from 1 day) also provided the exact time of each collection in their diary. They provided 2 mL of pure saliva (no cotton swab) in each saliva tube. Participants were instructed to store the samples in their refrigerator immediately until recollection by the research team (less than 5 days later). Upon recollection by the research team, saliva samples were stored in freezers at −20°C at the CSHS^1^ until assayed using a high-sensitivity enzyme immunoassay kit (Salimetrics®, State College, PA, Catalog No. 1-3,102). Frozen samples were brought to room temperature and centrifuged at 15,000 × *g* (3,000 rpm) for 15 min. The detection range for this assay is 0.012–3 μg/dL. When duplicate assay values were obtained for each sample, these values were averaged. Two measures of the diurnal cortisol pattern were used as outcomes: (1) the cortisol awakening response (CAR) and (2) the area under the curve with respect to ground (AUCg). The CAR was calculated as the percentage increase between the immediate wake-up cortisol level and the cortisol level 30 min after awakening (Clow et al., 2004). The AUCg, which captures the total diurnal cortisol secretion, was calculated with all four saliva samples using the formula described by Pruessner et al. (2003).

#### 3.4.2 Sociodemographic characteristics

Information was collected on sex, age, living situation, level of education, use of psychosocial care, and functional autonomy. First, we distinguished between participants living alone and those living with someone (0 = living with someone; 1 = living alone). The measure of educational level included three categories: 1 = elementary school, 2 = high school, and 3 = postsecondary. Psychological care overflow consisted of a self-report question asked at the 3-month follow-up in which participants were asked to indicate whether they had received any psychosocial care (i.e., psychological therapy, group therapy or support, follow-up with a social worker) during the course of the study. The variable is divided into two categories: (1) use of psychosocial care services during the study and (2) non-use.

Perceived functional limitations were assessed using the French short version of the Assessment of Life Habits (LIFE–H; Fougeyrollas et al., 1998). Based on the respondent’s perspective, the LIFE–H measures the accomplishment of life habits divided into eight categories: “daily activities” (e.g., communication [8 items], nutrition [4 items], housing [8 items], mobility [5 items], and personal care and health [8 items]) and “social roles” (e.g., responsibility [8 items], community and spiritual life [8 items], and recreation [7 items]). The purpose of the instrument was to identify the disabling situations experienced, taking into account the interaction between personal factors and environmental barriers that, in combination, resulted in a functional limitation. For each life habit, the respondent must indicate (a) its level of achievement (4 = without difficulty, 3 = with difficulty, 2 = achieved by substitution, 1 = not achieved, 0 = not applicable) and (b) the type of help required to achieve the life habit (4 = no help, 3 = with technical help, 2 = with adjustment, 1 = with someone’s help). To calculate a global score, each combination of responses between the two scales is assigned a weight from 0 to 9 and then normalized. A score of 10 indicates the absence of limitations in carrying out one’s life habits. The instrument has good psychometric properties with older adults (Desrosiers et al., 2004; Levasseur et al., 2008; Roy-Bouthot et al., 2014) and a reliability (Cronbach’s αs) of 0.85 in our sample.

### 3.5 Statistical analyses

Physiological and psychological variables were examined for outliers, defined as values ±3 standard deviations from the mean. Analyses were performed on Winsorized distributions. All physiological and psychological outcomes were continuous variables. To describe our sample, we calculated frequencies and means and performed cross-tabulations to observe the distribution of sociodemographic variables. We used *t*-tests and *χ*^2^ tests in univariable analysis to determine the equivalence of the experimental and control conditions at the pretest on the dependent variables.

To assess the effects of the intervention on coping strategies, mental health indicators, and cortisol levels, we conducted repeated-measures analyses of variance (RM ANOVAs) with measurement occasions as a within-subject factor and intervention as a between-group factor. Given the high attrition of distressed individuals at follow-up, two sets of analyses were conducted. The change in sample characteristics between posttest and follow-up supports this strategy. The first set of RM ANOVAs was conducted to compare the interaction between the conditions (SMT vs. waitlist) and the first two measurement times (pre-intervention and 3 weeks post-intervention) on each outcome. The second set included all three measurement occasions. This method is appropriate to account for within-subject dependencies between observations in the analysis (Singh et al., 2013). When data violated the assumption of sphericity as indicated by Mauchly’s test of sphericity, a Greenhouse–Geisser adjustment was performed. In addition, sociodemographic variables that were not equivalent between the conditions were included as covariates in all RM ANOVAs using a backward elimination method. In the first step, the model included all covariates; then, the variable with the highest *p*-value above 0.25 was removed, and the analysis was repeated with the remaining covariates. This procedure was repeated until the final model included only the covariate(s) contributing to the main effect with a *p*-value below 0.25 (Hosmer et al., 2013). This strategy was used to maintain statistical power and maximize the likelihood of detecting the main effects given the size of our sample. In all analyses, an alpha level of 0.05 (two-tailed) was used to indicate statistical significance. Cohen’s *d* was used to estimate effect size with the following interpretations describing critical values: small, *d* = 0.2; medium, *d* = 0.5; and large, *d* = 0.8. Data were analyzed with SPSS Statistics (version 25, IBM Corp., Armonk, NY).

#### 3.5.1 Attrition

A total of 62.9% of the sample completed all three measures (*n* = 107/170). The attrition rates were similar at the 3-week (25.9%) and the 3-month post-evaluations (28.8%). The most common reasons for attrition were (a) illness requiring isolation or hospitalization, (b) diagnosis of a serious illness (e.g., cancer), and (c) changes in caregiving responsibilities.

There was a significant difference in the attrition rate in the two group conditions at the posttest measure (Pearson *χ*^2^ = 4.45, *p* = 0.035), with more missing data than expected in the experimental group. No difference was found at the 3-month follow-up (Pearson *χ*^2^ = 0.206, *p* = 0.650). The baseline characteristics of all study participants, those lost to follow-up at 3 months, and those who remained in the study are presented in Supplementary Table 6 (Supplementary Appendix). In SPSS, all analyses excluded participants with missing data, so each RM ANOVA was performed listwise on the included variables.

## 4 Results

### 4.1 Baseline descriptive statistics

Sociodemographic characteristics data for the control and experimental groups and for all participants are shown in Table 2. The sample was 85.6% female, and 59% lived alone. Seven percent had an elementary school education, 62.8% had a high school diploma, and almost 30% had a college or university degree. Nearly one-fifth of the participants reported receiving some type of psychological care during the course of the study. These services could include individual or group therapy, group support, or follow-up by social workers or other mental health professionals. At baseline, the two groups did not differ on these demographic variables. However, comparisons of the means and the proportions between the two groups, calculated using *t*-tests and *χ*^2^s, showed significant differences in age and functional autonomy. Participants in the SMT group were significantly younger (*M* = 74.71 years, *SD* = 8.28) than those in the waitlist group (*M* = 77.72 years, *SD* = 6.57), *t* (164) = −2.688, *p* = 0.008. The magnitude of the mean differences was small to medium. Functional autonomy was significantly different, and this was a medium-sized effect. Therefore, age and functional autonomy were included as covariates in the subsequent analyses. Finally, at baseline, the two groups did not differ on any of the dependent variables (*t-*test results are shown in Table 3).

**Table 2:** Frequencies and chi-square and t-test results for sociodemographic characteristics by group assignment and for the entire sample.

Sociodemographic characteristics	Full sample (*N* = 170)		SMT (*n* = 90)		Control (*n* = 80)			
	*n*	%	*n*	%	*n*	%	*χ*^2^ (*df*)	
Sex							0.586 (1)	
Female	145	85.3	75	83.3	70	87.5		
Male	25	14.7	15	16.7	10	12.5		
Cohabiting	68	41.0	32	36.4	36	46.2	1.639 (1)	
Highest education level							1.882 (2)	
Middle school	12	7.3	7	8	5	6.5		
High school	103	62.8	58	66.7	45	58.4		
Postsecondary	49	29.9	22	25.3	27	35.1		
Psychosocial care	28	23.0	17	25.8	11	19.6	0.641 (1)	
	*M*	*SD*	*M*	*SD*	*M*	*SD*	*t* (*df*)	Cohen’s *d*
Age	76.07	7.67	74.61	8.28	77.72	6.57	−2.651 (164)*	0.40
FA	9.15	0.92	9.37	0.67	8.91	1.09	3.125 (152)**	0.50

SMT, stress management training; control, waitlist control group; FA, functional autonomy. *p < 0.05. **p < 0.005.

**Table 3:** Results of t-tests for coping style, mental health, and cortisol variables according by group at baseline.

Outcome	Full sample		SMT		Control			Cohen’s *d*
	*M*	*SD*	*M*	*SD*	*M*	*SD*	*t* (*df*)	
Coping style								
SP	2.73	0.54	2.72	0.48	2.73	0.59	−0.07 (157)	0.02
Proactive	2.74	0.33	2.74	0.35	2.75	0.32	−0.23 (158)	0.03
Mental health								
Stress	1.64	0.63	1.66	0.60	1.62	0.65	0.41 (160)	0.06
Anxiety	1.28	0.62	1.31	0.60	1.25	0.64	0.61 (161)	0.10
Depression	0.73	0.48	0.74	0.47	0.73	0.49	0.23 (162)	0.10
Cortisol								
CAR	35.25	92.95	40.94	107.46	28.59	72.63	0.82 (152)	0.14
AUCg	2.03	1.09	2.03	1.05	2.03	1.15	−0.03 (143)	0.00

Mean parameter values for each of the analyses are shown for the SMT condition (*n* = 90) and the waitlist (control) condition (*n* = 80), as well as the results of the *t*-tests (assuming equal variance) comparing the parameter estimates between the two groups. SMT, stress management training; CAR, cortisol awakening response; SP, strategic planning; AUCg, area under the curve with respect to the ground.

### 4.2 Effects of SMT on coping strategies, mental health, and cortisol at the 3-week posttest evaluation

**Figure 2:**
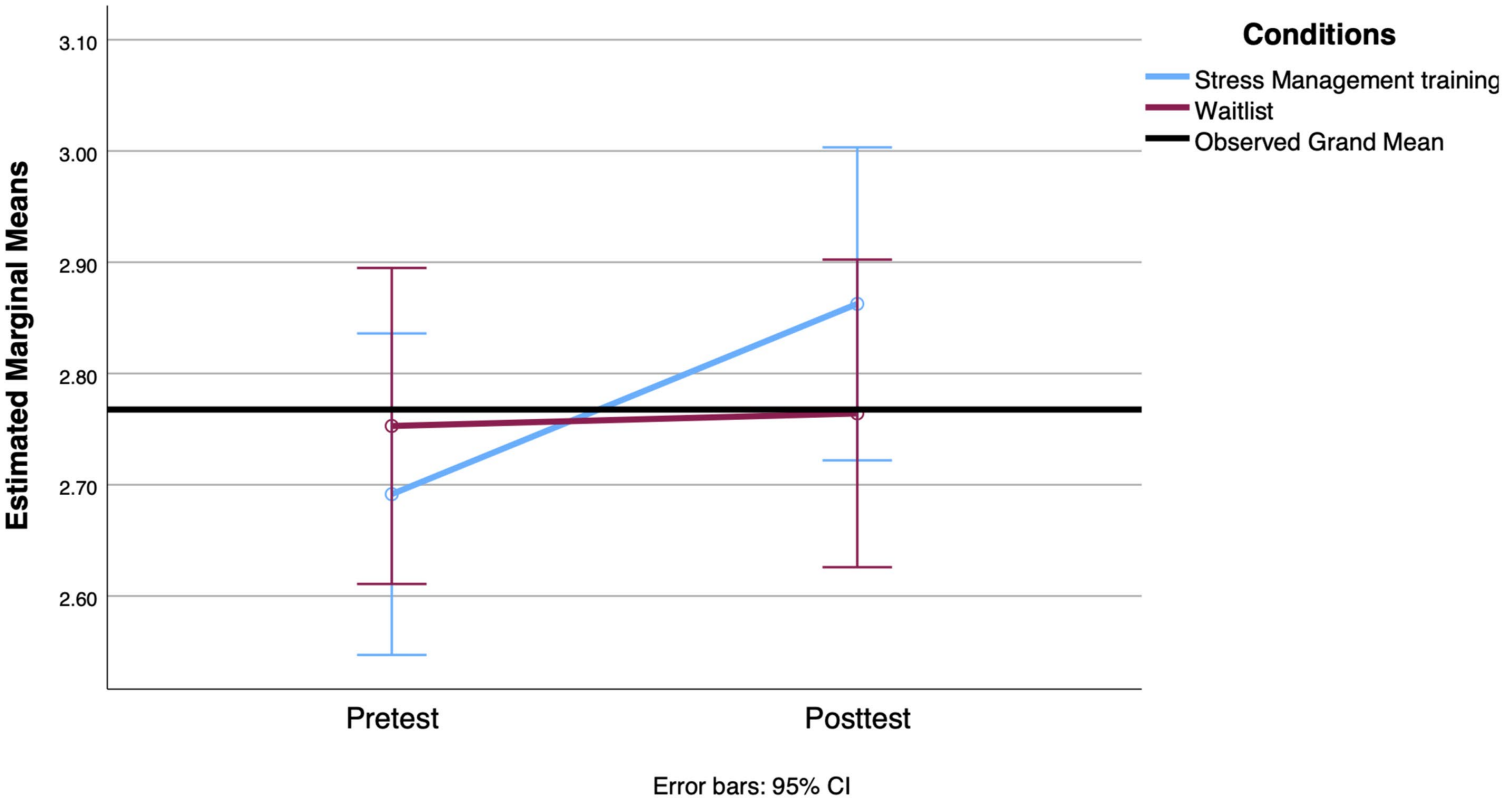
Mean change observed between groups in strategic planning from the pretest to the 3-week posttest.

**Figure 3:**
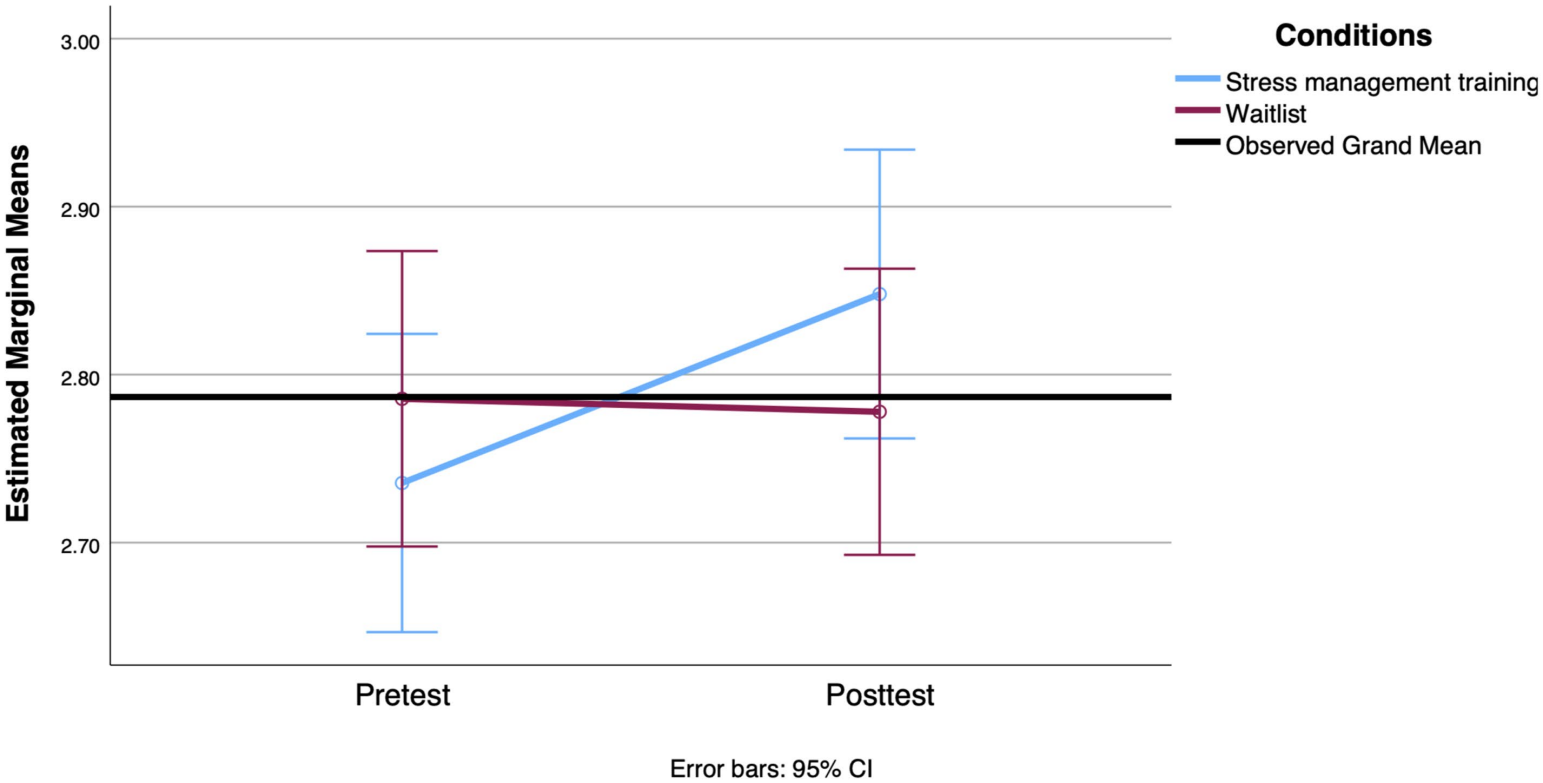
Mean change observed between groups in proactive coping from the pretest to the 3-week posttest.

We conducted the first set of RM ANOVAs to examine the effect of the SMT intervention compared to the waitlist condition on participants’ scores on coping styles (strategic planning and proactive coping), mental health (stress, anxiety, and depression), and cortisol levels (awakening response and AUCg) across the two time points (pre-intervention and 3-week posttest). The means and standard deviations of all outcomes assessed at pre- and post-treatment are shown in Table 4. For the coping style outcome, the results of the RM ANOVA indicated that the interaction between group and time was statistically significant when comparing strategic planning scores [*F*(1, 114) = 4.209, *p* = 0.043, Cohen’s *d* = 0.39]. The means indicate a more pronounced increase in the use of these strategies reported by participants in the SMT group, with a small effect size. However, both groups showed an increase in the strategic planning scale over time [*F*(1, 114) = 5.484, *p* = 0.021, Cohen’s *d* = 0.44]. With regard to proactive coping, the backward elimination method left functional autonomy as a covariate. However, it was not significantly related to time [*F*(1, 110) = 1.507, *p* = 0.222, Cohen’s *d* = 0.24]. After adjusting for this covariate, there was a significant effect between proactive coping over time and group [*F*(1, 110) = 6.250, *p* = 0.014, Cohen’s *d* = 0.48], with the proactive coping score increasing more over time for the SMT participants. Figures 2, 3 show the profile plot for both coping strategies.

**Table 4:** Means, standard deviations, and RM ANOVA results between the pretest and the 3-week posttest on coping strategies, mental health, and cortisol indicators.

Variable	SMT		Control			RM ANOVA		
	*M*	*SD*	*M*	*SD*	Effect	*F*(*df*)	*p*	Cohen’s *d*
Strategic planning								
*n*	57		59		T	5.484 (1,114)	0.021	0.44
Pretest	2.69	0.50	2.75	0.59	T × FA	—	*ns*	—
Posttest	2.86	0.43	2.76	0.62	T × Age	—	*ns*	—
					T × G	4.209 (1,114)	0.043	0.39
Proactive coping								
*n*	56		57		T	2.110 (1, 110)	0.149	0.28
Pretest	2.76	0.34	2.76	0.33	T × FA	1.507 (1,110)	0.222	0.24
Posttest	2.86	0.32	2.81	0.32	T × Age	—	*ns*	—
					T × G	6.250 (1,110)	0.014	0.48
Stress								
*n*	54		61		T	3.777 (1,112)	0.054	0.37
Pretest	1.62	0.61	1.57	0.62	T × FA	2.109 (1, 112)	0.149	0.27
Posttest	1.34	0.52	1.41	0.59	T × Age	—	*ns*	—
					T × G	2.998 (1,112)	0.086	0.33
Anxiety								
*n*	56		61		T	8.209 (1, 114)	0.005	0.54
Pretest	1.24	0.64	1.18	0.60	T × FA	6.121 (1, 114)	0.015	0.46
Posttest	1.05	0.54	1.08	0.53	T × Age	—	*ns*	—
					T × G	4.165 (1, 114)	0.044	0.38
Depression								
*n*	59		63		T	4.998 (1, 119)	0.027	0.41
Pretest	0.66	0.42	0.72	0.46	T × FA	—	*NS*	—
Posttest	0.62	0.43	0.67	0.43	T × Age	4.474 (1, 119)	0.036	0.39
					T × G	0.385 (1, 119)	0.536	0.11
CAR								
*n*	69		56		T	4.524 (1, 123)	0.035	0.38
Pretest	43.91	114.69	32.86	69.71	T × FA	—	*ns*	—
Posttest	18.64	62.68	21.56	57.19	T × Age	—	*ns*	—
					T × G	0.539 (1, 123)	0.464	0.13
AUCg								
*n*	54		57		T	10.081 (1, 98)	0.002	0.64
Pretest	2.03	0.99	2.15	1.27	T × FA	8.960 (1, 98)	0.003	0.61
Posttest	1.65	0.77	2.06	1.10	T × Age	—	*ns*	—
					T × G	4.546 (1, 98)	0.035	0.43

Covariates were entered into the analysis using a backward elimination strategy. Only data from the final model data are included in the table. RM ANOVA, repeated-measures analysis of variance; SMT, stress management training; control, waitlist control group; CAR, cortisol awakening response; AUCg, area under the curve with respect to ground; G, group; T, time; FA, functional autonomy.

**Figure 4:**
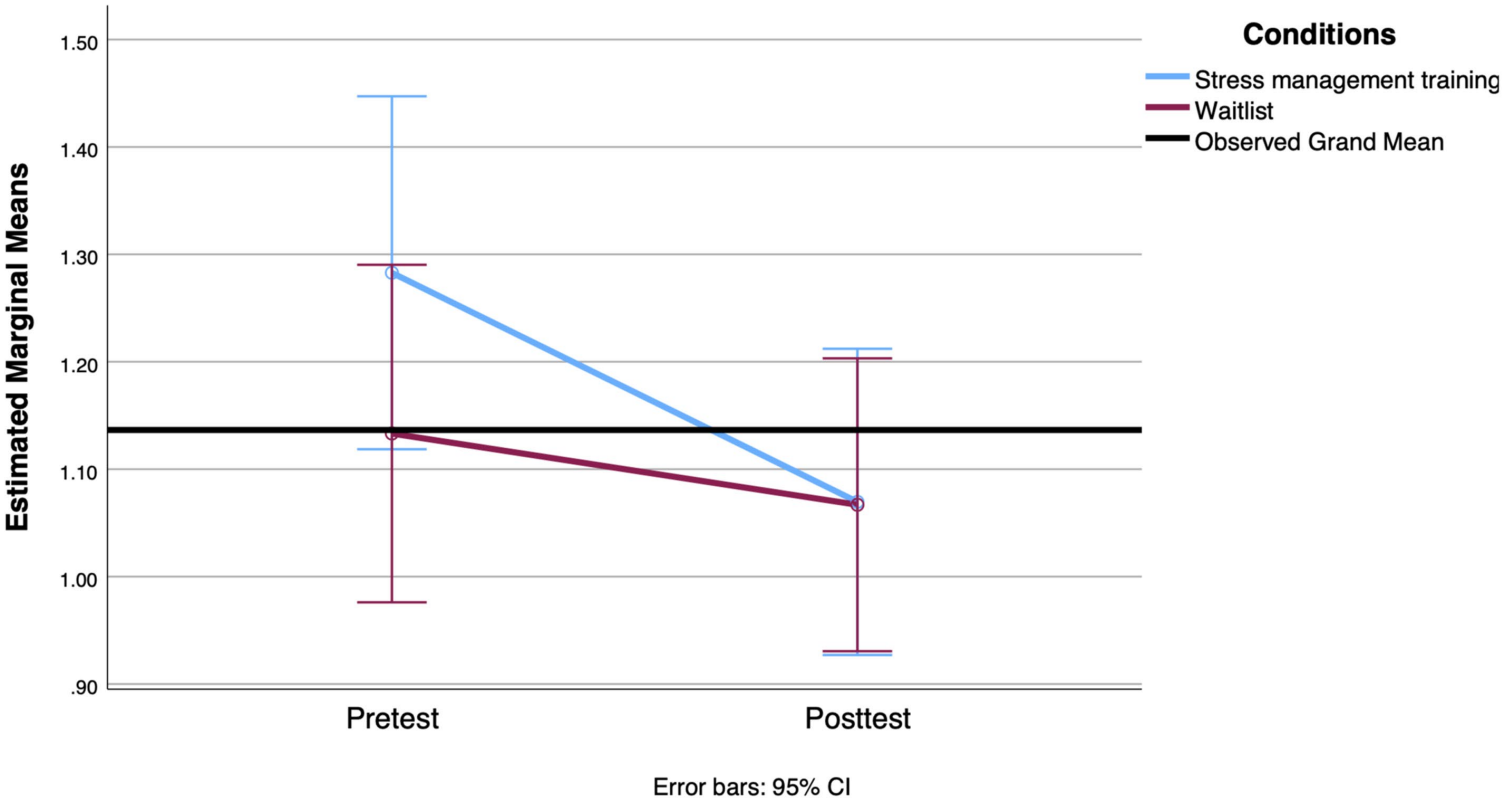
Mean change observed between groups on the anxiety scale from the pretest to the 3-week posttest.

For mental health outcomes, there was no significant interaction between group and time for perceived stress [*F*(1, 112) = 2.998, *p* = 0.086, Cohen’s *d* = 0.33] and depression scores [*F*(1, 119) = 0.385, *p* = 0.536, Cohen’s *d* = 0.11]. For depression scores, the results showed a significant effect of time [*F*(1, 119) = 4.998, *p* = 0.027, Cohen’s *d* = 0.41] and a significant interaction between time and age [*F*(1, 119) = 4.474, *p* = 0.036, Cohen’s *d* = 0.39]. A decrease in mean depression scores was observed in both groups. In addition, younger age was associated with greater reductions in depression scores over time. Analyses conducted on anxiety revealed a significant main effect of time [*F*(1, 114) = 8.209, *p* = 0.005, Cohen’s *d* = 0.54] and functional autonomy [*F*(1, 114) = 6.121, *p* = 0.015, Cohen’s *d* = 0.46] as covariates, but also a significant interaction between time and group [*F*(1, 114) = 4.165, *p* = 0.044, Cohen’s *d* = 0.38]. This interaction, suggesting that the decrease in anxiety from the first to the second measurement was more pronounced in the SMT group, is shown in Figure 4. The decrease in anxiety score was associated with lower functional autonomy in both groups combined.

**Figure 5:**
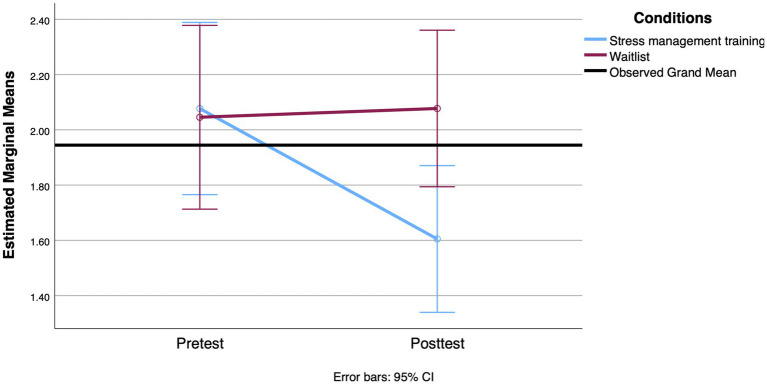
Mean change in AUCg value observed between groups from the pretest to the 3-week posttest.

For the physiological outcomes, although the interaction effect between group and time on the CAR values was not significant [*F*(1, 98) = 0.539, *p* = 0.464, Cohen’s *d* = 0.13], the main effect of time was significant [*F*(1, 91) = 4.524, *p* = 0.035, Cohen’s *d* = 0.38]. The results showed a decrease in the mean CAR in both groups. On the other hand, there was a significant main effect of the interaction of group and time on the AUCg values with a small effect size [*F*(1, 91) = 4.986, *p* = 0.028, Cohen’s *d* = 0.43]. Participants in the SMT group showed a more significant decrease in daily cortisol levels over time (see Figure 5). This result was adjusted for the level of functional autonomy, which showed a significant interaction with time [*F*(1, 98) = 8.960, *p* = 0.003, Cohen’s *d* = 0.61]. A greater decrease in AUCg values over time was associated with lower functional autonomy.

### 4.3 Effects of SMT on coping strategies, mental health, and cortisol at the 3-month follow-up

The second set of RM ANOVAs examined the differences between the three measurement occasions on the same outcomes. Although the interaction effect between group and time on the strategic planning scores was not significant [*F*(2, 196) = 1.809, *p* = 0.166, Cohen’s *d* = 0.27], the main effect of time was significant [*F*(2, 196) = 3.831, *p* = 0.023, Cohen’s *d* = 0.40]. After adjusting for participant age, the interaction between group and time was significant in regard to the proactive coping strategies scores [*F*(2, 192) = 3.195, *p* = 0.043, Cohen’s *d* = 0.36], with a small effect size. However, an examination of the mean scores at each measurement occasion for both groups revealed an increase in reported use of proactive coping strategies after the intervention for the SMT group, followed by a decrease close to the baseline 3 months after the intervention (see Table 5).

**Table 5:** Means, standard deviations, and RM ANOVA results between pretest and follow-up on coping strategies, mental health, and cortisol indicators.

Variable	SMT		Control			RM ANOVA		
	*M*	*SD*	*M*	*SD*	Effect	*F* (*df*)	*p*	Cohen’s *d*
Strategic planning								
*n*	52		48		T	3.831 (2,196)	0.023	0.40
Pretest	2.67	0.51	2.76	0.60	T × FA	—	*ns*	—
Posttest	2.85	0.43	2.79	0.67	T × Age	—	*ns*	—
Follow-up	2.79	0.44	2.81	0.54	T × G	1.809 (2,196)	0.166	0.27
Proactive coping								
*n*	52		47		T	1.409 (2, 192)	0.247	0.24
Pretest	2.73	0.34	2.78	0.36	T × FA	—	*ns*	—
Posttest	2.83	0.33	2.77	0.36	T × Age	1.409 (2,192)	0.247	0.24
Follow-up	2.74	0.32	2.80	0.35	T × G	3.195 (2, 192)	0.043	0.36
Stress								
*n*	49		50		T	4.298 (2, 190)	0.015	0.42
Pretest	1.67	0.59	1.54	0.63	T × FA	2.807 (2, 190)	0.063	0.35
Posttest	1.36	0.53	1.36	0.58	T × Age	—	*ns*	—
Follow-up	1.37	0.54	1.42	0.55	T × G	3.750 (2, 190)	0.025	0.40
Anxiety								
*n*	51		50		T	2.977 (2, 196)	0.053	0.35
Pretest	1.29	0.63	1.10	0.55	T × FA	2.326 (2, 196)	0.100	0.31
Posttest	1.10	0.52	1.03	0.48	T × Age	—	*ns*	—
Follow-up	1.11	0.48	1.08	0.53	T × G	2.347 (2, 196)	0.098	0.31
Depression								
*n*	54		51		T	2.465 (2, 204)	0.088	0.31
Pretest	0.69	0.42	0.67	0.45	T × FA	—	*ns*	—
Posttest	0.65	0.42	0.64	0.44	T × Age	2.300 (2, 204)	0.103	0.30
Follow-up	0.63	0.43	0.62	0.44	T × G	0.105 (2, 204)	0.901	0.06
CAR								
*n*	53		44		T	2.255^a^ (2, 186)	0.108	0.31
Pretest	53.29	126.61	36.45	74.86	T × FA	1.409 ^a^ (2, 186)	0.247	0.25
Posttest	20.22	67.95	25.27	61.12	T × Age	1.642 ^a^ (2, 186)	0.198	0.26
Follow-up	36.33	64.34	52.21	83.83	T × G	1.261 ^a^ (2, 186)	0.285	0.23
AUCg								
*n*	44		41		T	1.999 ^a^ (2, 162)	0.139	0.31
Pretest	2.04	0.97	2.04	1.18	T × FA	2.243 ^a^ (2, 162)	0.109	0.33
Posttest	1.78	0.71	2.13	1.15	T × Age	1.448 ^a^ (2, 162)	0.238	0.27
Follow-up	2.05	0.84	2.56	1.46	T × G	3.604 ^a^ (2, 162)	0.029	0.42

Covariates were entered into the analysis using a backward elimination strategy. Only data from the final model are included in the table. RM ANOVA, repeated-measures analysis of variance; SMT, stress management training; control, waitlist control group; CAR, cortisol awakening response; AUCg, area under the curve with respect to ground; G, group; T, time; FA, functional autonomy. ^a^Greenhouse–Geisser corrected.

**Figure 6:**
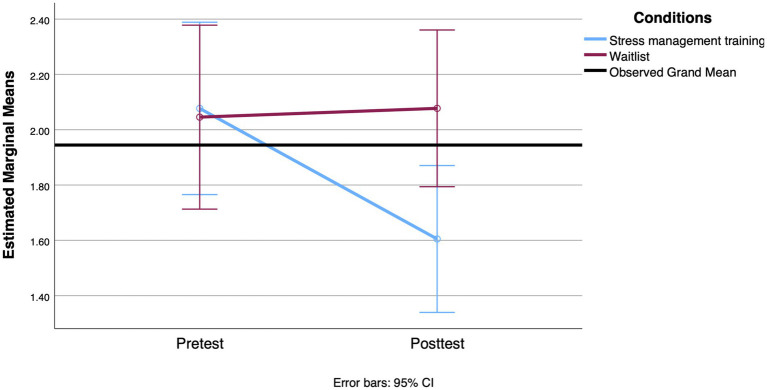
Mean change between groups on the perceived stress scale from the pretest to the 3-month follow-up.

For mental health outcomes, there was no significant time-by-group interaction for anxiety [*F*(2, 196) = 2.347, *p* = 0.098, Cohen’s *d* = 0.31] or depression scores [*F*(2, 204) = 0.105, *p* = 0.901, Cohen’s *d* = 0.06]. For perceived stress, the analysis revealed that the control group appeared to deteriorate between the second and third measures, while the intervention group’s mean score remained stable [*F*(2, 190) = 3.750, *p* = 0.025, Cohen’s *d* = 0.04; see Figure 6]. The result is adjusted for the level of autonomy.

**Figure 7:**
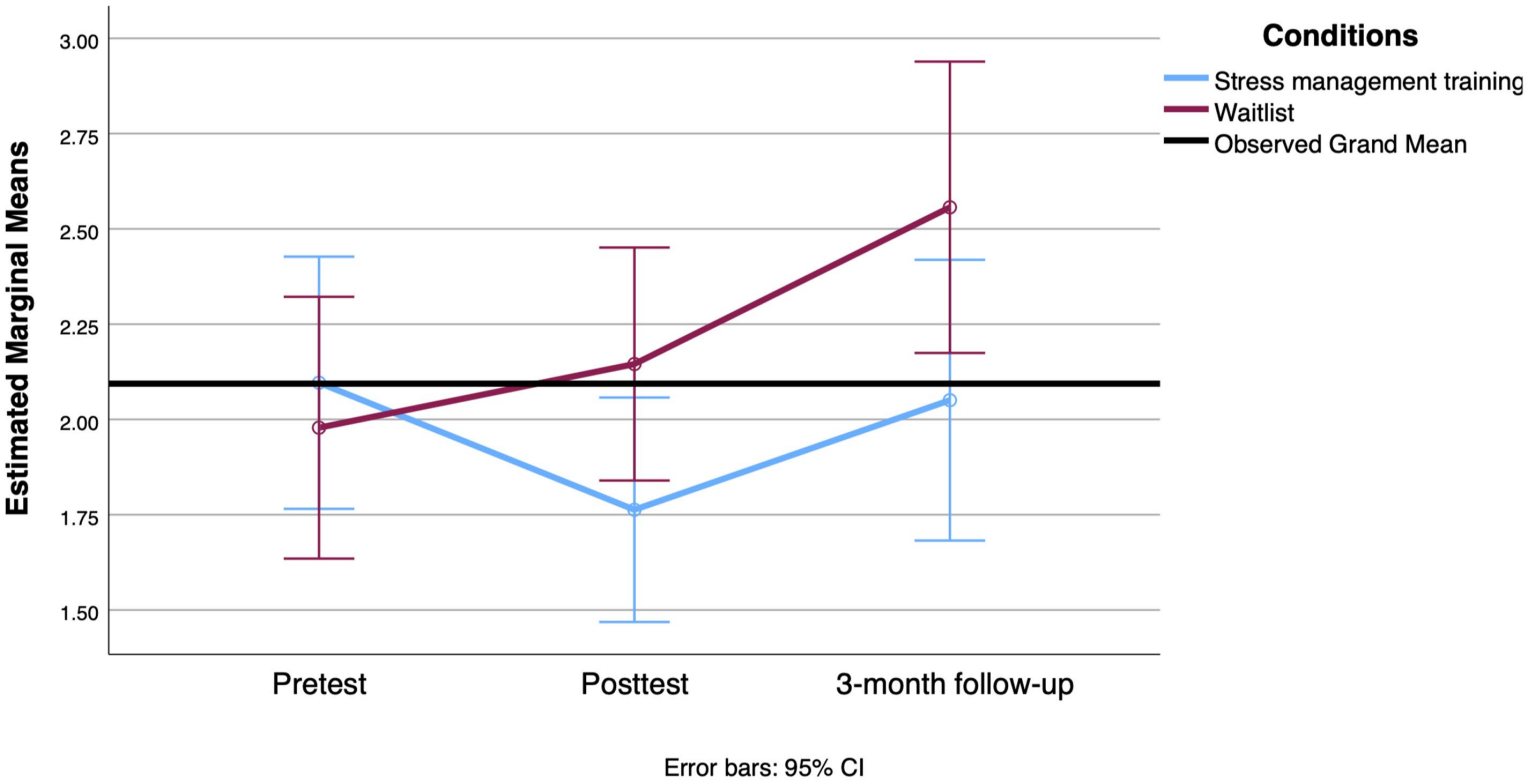
Mean change observed between groups at the AUCg level from the pretest to the 3-month follow-up.

The difference between the intervention and control groups reached significance at the follow-up for AUCg when adjusted for the participants’ level of functional autonomy and age. There was a significant interaction between group and time with a small effect size [*F*(2, 162) = 3.604, *p* = 0.029, Cohen’s *d* = 0.23]. Figure 7 shows a small decrease in cortisol released during the day at the posttest only in the SMT group, followed by an increase at the 3-month follow-up. The tests revealed no significant differences in the interaction term between treatment group and time on the CAR [*F*(2, 186) = 1.261, *p* = 0.285, Cohen’s *d* = 0.23, Greenhouse–Geisser corrected].

## 5 Discussion

In this study, we examined the effects of a 6-week group-based stress management intervention with a stress contagion component. The intervention was adapted to meet the needs and characteristics of older adults.

First, 3 weeks after the intervention, we found a significant increase in the use of problem-solving coping strategies among participants in the experimental condition compared to the waitlist condition. Specifically, participants reported using more proactive and strategic planning coping styles after the intervention. Proactive coping includes strategies used to prevent or minimize the impact of future stressors (e.g., taking the initiative to solve a problem). Greenglass et al. (2006) found that when individuals are able to identify signs of a potential or imminent threat and have acquired skills to cope with it (e.g., planning and goal-setting), they are more likely to feel a sense of control that leads them to take adaptive actions. In a study that examined the relationship between proactive coping, daily hassles, functional disability, and other health factors in a sample of 224 community-dwelling older adults, the authors found that the use of proactive coping positively affected participants’ independence and well-being by contributing to the completion of daily tasks (Fiksenbaum et al., 2006). Our results suggest that the intervention supported the acquisition of these problem-solving skills. The development of these strategies was also among the most effective core components of meta-analyses of stress management interventions (Chiesa and Serretti, 2009; Querstret et al., 2020; Richardson and Rothstein, 2008). We postulate that, in addition to the intervention components, the group aspect may have positively contributed to our findings. Indeed, studies have demonstrated the positive influence of social support on the increased use of coping strategies (Fiksenbaum et al., 2006; Lee, 2018; Trouillet et al., 2009). The group intervention was tailored to provide participants with opportunities to talk about their stressors and to support each other. While the SMT intervention demonstrated significant improvements in proactive and strategic planning coping strategies at the 3-week posttest, these effects did not persist at the 3-month follow-up. Notably, both proactive coping strategies and strategic planning scores returned closer to baseline levels, suggesting that the initial gains from the intervention diminished over time. This decline could be attributed to several factors. First, the intervention’s impact may have been context-specific or short-lived, requiring ongoing reinforcement to maintain its effectiveness. Additionally, the absence of sustained effects may reflect the natural variability in individual coping practices or the possible lack of long-term integration of the learned strategies into daily routines. We recall that the effects of our SMT conditions were demonstrated against a waitlist condition, suggesting that we cannot disentangle the effects of the SMT component from the support group.

Second, our results showed significant effects on two measures of mental health. Exposure to the program appeared to result in decreased feelings of anxiety at 3 weeks postintervention and decreased perceived stress at the 3-month follow-up. It is notable, however, that while anxiety improved shortly after the intervention, these gains were not sustained over the longer term, as there was no significant change in anxiety levels at the 3-month mark. The paucity of research on CBT stress management interventions for older adults has obscured possible comparisons (Hofmann et al., 2012; Li and Bressington, 2019). A meta-analysis of seven randomized controlled trials comparing the efficacy of CBT (including group therapy) for late-life anxiety disorders with control conditions found significant reductions in anxiety, worry, and depressive symptoms after the intervention (Hendriks et al., 2008). In comparison, our program, that is shorter with its six sessions, suggested promising results on anxiety and stress in the short term (after 3 weeks and 3 months, respectively). The lack of sustained improvement in anxiety and the small differences in our psychological outcomes at the 3-month follow-up are in line with other experimental studies with older adults in which gains on stress, anxiety, or depression scales were rarely maintained (Hofmann et al., 2012; Li and Bressington, 2019). These findings may be due to the short duration of our intervention, coupled with the need for a sustained effort to form new habits for coping with stressors. Additionally, numerous adaptive challenges may emerge in older individuals, potentially limiting the long-term effectiveness of otherwise successful treatments and impacting their ability to maintain newly acquired skills without ongoing support.

The third finding of the present study concerns our program’s effect on the participants’ cortisol levels. The mean CAR was calculated to represent the increase in cortisol concentration that occurs in the first half-hour after awakening. Typically, the increase in glucocorticoids is about 50 to 75% and is thought to prepare individuals to engage with the environment to face the day ahead (Fries et al., 2009). No association was found between both groups of participants and changes in CAR over time. It should be noted that, at baseline, only 32.62% of our sample achieved an increase in cortisol concentration of at least 50%, suggesting lower cortisol levels upon awakening. However, it should be noted that we also found a large interindividual variability in the CAR results. The participants were diligent in filling in their saliva diaries, so we have access to the collection times for each sample included in the study. One possible explanation of those results is the duration required to collect the sample—information that was not compiled. For example, participants mentioned that xerostomia—a side effect of medication—caused complications in providing the 2 mL of saliva promptly. However, the size of our sample did not allow us to control for the many variables affecting CAR that could have addressed these concerns, such as smoking, illness, and medication, to name a few (Stalder et al., 2016; Stalder et al., 2022).

In contrast, participants in the SMT group showed a significant reduction in AUCg 3 weeks after the intervention, whereas cortisol levels increased in the control group. Exposure to chronic stress has been shown to alter diurnal cortisol patterns at any age (for a review, see Gaffey et al., 2016). In the context of age-related changes in the HPA axis, resilience to stress would be enhanced by the development of individual resources such as emotional regulation and social support. Conversely, a lack of these resources could exacerbate HPA axis alterations. More specifically, based on available theoretical data, the aging, stress, and resilience model (Gaffey et al., 2016) proposes that social support and coping style influence diurnal cortisol in aging. For example, older adults who experienced greater social strain from their network had elevated evening cortisol and a flatter cortisol slope across the day (Friedman et al., 2012). Rueggeberg et al. (2012) demonstrated that coping strategies also have an impact on AUCg. Their study highlighted that ill individuals who used more self-protective strategies (i.e., cognitive strategies to reevaluate a situation and to avoid self-blame) had lower AUCg when reporting loneliness. In other words, in the presence of adversity, the use of certain cognitive strategies to cope with stressors has a positive impact on diurnal cortisol. Our program sought to influence these very determinants: it aimed to teach coping strategies to reevaluate stressors through a psychoneuroendocrine lens. It also promoted strategies to enhance social support while providing emotional and instrumental support from the group modality. Although the effect size was small, the main effect found in diurnal cortisol concentration is promising. Few studies have used physiological measures to assess the efficacity of stress management interventions. At the 3-month follow-up, cortisol levels in the SMT group showed a rebound, with AUCg values returning closer to baseline levels. However, it’s important to note that despite this rebound, there was still a significant effect of the SMT intervention on AUCg values at the 3-month mark, although the effect size was smaller. This suggests that while the initial benefits of the intervention on cortisol regulation were not fully sustained over time, some residual effects remained. This finding aligns with other research indicating that short-term improvements in physiological measures can diminish if not continuously supported (Gaffey et al., 2016). The intervention’s decrease in effectiveness over time might be due to the absence of ongoing reinforcement or follow-up, as well as the challenges older adults face in maintaining stress management techniques long-term.

Given that few studies have used physiological measures to assess the efficacy of stress management interventions, our results contribute valuable insights into how such programs can impact cortisol regulation. The observed rebound in AUCg, along with the reduced but still present effect at 3 months, underscores the need for future research to explore ways to extend the benefits of stress management interventions and maintain their physiological impacts over longer periods.

Our study has several limitations. The first is the lack of proportionality in the recruitment in the specific settings chosen (i.e., high-end PRs, services for older caregivers, and day centers). The sample includes a higher proportion of people living in high-end PRs, which are not representative of most seniors in the general population: in Québec, 17% of older adults live in PRs; in our study, they represented about 75% (SCHL, 2021). This limits the generalizability of the results. However, this population is rarely studied because of its presumed socioeconomic privilege. Nevertheless, our results showed a significantly higher rate of distress in our sample compared to the rates found in other studies. The proportion of participants above the clinical threshold on the total HADS scale (score ≥ 16) in our sample was high: 43.9%. Several reasons may explain the observed levels of distress. Some participants were juggling multiple responsibilities, including caregiving and supporting family members. Others had just moved into their new homes because many residences opened just weeks before the study began. For some, the move was prompted by a serious health problem, a partner’s loss of autonomy, or other stressful situations. This suggests that the recruitment location was important in identifying at-risk individuals who could benefit from the intervention. Future research should examine other populations of older adults to determine the generalizability of the findings.

Second, although the attrition rate at the postintervention assessment and at the 3-month follow-up may seem high, it is consistent with what is typically seen in studies of older people testing the efficacy of a treatment. The attrition rate was not different between our two groups: 25.9 and 28.8%, respectively. A total of 62.9% of the sample responded to all three measures. Participants who dropped out by the time of the follow-up had significantly higher levels of combined anxiety and depressive symptoms at baseline. Although this is common in geriatric research, it can introduce significant bias into the results and affect the magnitude of the findings. In a systematic review of the literature on attrition among older adults in longitudinal studies, Chatfield et al. (2005) reported attrition rates ranging from 2 to 50% between measurement waves. In studies of older adults with chronic conditions, attrition rates as high as 30% are not uncommon (Chatfield et al., 2005; Gardette et al., 2007). Although we initially recruited 170 participants, which is generally an adequate number for a two-arm CRT, analysis of physiological data—in particular, cortisol—often requires even more participants. Therefore, we may have lacked statistical power.

Although this study has some limitations, it is important to note that the *O’stress* program is a brief preventive intervention program focused on stress and stress contagion that appears to increase coping strategies and decrease levels of anxiety and perceived stress. *O’stress* also appeared to reduce participants’ diurnal cortisol levels, which has not often been demonstrated in previous studies. In fact, our study is one of the few to demonstrate the efficacy of a stress management intervention in older adults on both subjective and objective measures of stress. However, some of these effects on coping and anxiety were not maintained over time, highlighting the importance of adding booster sessions. Booster sessions review the procedures and strategies learned during the main intervention and are used to increase the magnitude or persistence of the intervention’s benefits. Given that, similar to our findings, the effects of most resilience programs in older adults tend to diminish over time, it seems essential to plan booster sessions in adavance, especially for short interventions. These booster sessions would be beneficial because the intervention requires the development of new habits, which can be time consuming. Furthermore, aging is generally not a period of stability, as older adults often face frequent adaptive challenges, which may contribute to the limited long-term effectiveness of otherwise impactful treatments.

We should emphasize that our results suggest that this type of brief preventive program could reduce the levels of basal cortisol in older adults, which could represent a significant protective factor against health outcomes that has been related to HPA function. The program, which was offered in a preventive approach in community settings to all older adults interested, had significant effects on a nonclinical sample. A larger implementation of the program could be ensured by training psychosocial workers and even peers in the community. This approach is used for the dissemination of *DeStress for Success©,* with a training session offered twice a year, and thousands of kids and teenagers have been able to benefit from the program.

Increasing life expectancy challenges us to find solutions to ensure that these extra years are synonymous with health and well-being. Such interventions, which take little time to implement and appear promising, have the advantage of being scalable. For example, the *DeStress for Success©* program, developed for adolescents, has already been distributed to more than 3,000 practitioners and teachers for use with their students. We believe that our study provides sufficient evidence to warrant further research to improve the effectiveness of *O’stress* in different settings. In addition, we plan to adapt the stress contagion component to older adults’ formal and informal networks. We argue that improving resilience to stress in aging should become part of a universal prevention intervention and be embedded in every setting that provides services to older people.

## Author’s note

This article is part of MJR’s thesis work.

## Data Availability

The raw data supporting the conclusions of this article will be made available by the authors, without undue reservation.

## Footnotes

^1^ www.humanstress.ca
