# Supplementary material for: Increasing stress resilience in older adults through a 6-week prevention program: effects on coping strategies, anxiety symptoms, and cortisol levels
Source: Front Psychol. 2025 Jan 6;15:1499609. doi: 10.3389/fpsyg.2024.1499609 (PMC11743716; doi:10.3389/fpsyg.2024.1499609)
Supplement: Supplementary file 1 [file Table_1.pdf]

Supplementary Material

Increasing stress resilience in older adults through a 6-week prevention program: effects on coping strategies, anxiety symptoms, and cortisol levels

Richer Marie-Josée, Grenier Sébastien, Lupien Sonia, Plusquellec Pierrich

*** Correspondence:** Marie-Josee Richer: marie-josee.richer@umontreal.ca

Table A.1. Baseline characteristics of all participants in the study, those lost at the 3-month follow-up, and those remaining in the study to the end.

Sociodemographic characteristics	All participants		Participants who dropped out by the 3-month follow-up		Participants still in the study at the 3-month follow-up	
	SMT (*n* = 90)	Control (*n* = 80)	SMT (*n* = 22)	Control (*n* = 22)	SMT (*n* = 68)	Control (*n* = 58)
Age (years), *M* (*SD*)	74.61 (8.3)	77.72 (6.6)	73.68 (9.2)	79.19 (6.8)	74.91 (8.0)	77.18 (6.5)
Gender						
Female	75 (83.3)	70 (68.2)	18 (81.8)	19 (86.4)	57 (83.8)	51 (87.9)
Male	15 (16.7)	10 (11.8)	4 (18.2	3 (13.6)	11 (16.2)	7 (12.1)
Cohabiting	32 (36.4)	36 (46.2)	6 (28.6)	7 (33.3)	26 (38.6)	29 (50.9)
Highest education level						
Middle school	7 (8.0)	5 (6.5)	1 (4.8)	3 (15.0)	6 (9.1)	2 (3.5)
High school	58 (66.7)	45 (58.4)	16 (76.2)	10 (50.0)	42 (63.6)	35 (61.4)
Postsecondary	22 (25.3)	27 (35.1)	4 (19)	7 (35.0)	18 (27.3)	20 (35.1)
Income (CAD)						
<20,000	11 (14.7)	7 (10.1)	3 (16.7)	1 (6.3)	8 (14)	6 (11.3)
21,000–40,000	35 (46.7)	16 (23.2)	5 (27.8)	5 (31.3)	30 (52.6)	11 (20.8)
41,000–60,000	21 (28.0)	19 (27.5)	9 (50.0)	4 (25.0)	12 (21.1)	15 (28.3)
61,000–80,000	4 (5.3)	9 (13.0)	1 (5.6)	2 (12.5)	3 (5.3)	7 (13.2)
81,000–100,000	3 (4.0)	6 (8.7)	0 (0.0)	0 (0.0)	3 (5.3)	6 (11.3)
>101,000	1 (1.3)	12 (17.4)	0 (0.0)	4 (25.0)	1 (1.8)	8 (15.1)
Use of psychosocial care	17 (25.8)	11 (19.6)			17 (25.8)	11 (19.6)
Stress, *M* (*SD*)	1.66 (0.60)	1.62 (0.65)	1.72 (0.68)	1.68 (0.60)	1.64 (0.58)	1.60 (0.67)
Anxiety, *M* (*SD*)	1.31 (0.60)	1.25 (0.64)	1.34 (0.61)	1.51 (0.67)	1.30 (0.60)	1.15 (0.60)
Depression, *M* (*SD*)	0.74 (0.47)	0.73 (.049)	0.86 (0.52)	0.81 (0.46)	0.70 (0.45)	0.69 (0.49)
CAR, *M* (*SD*)	40.94 (107.46)	28.59 (72.63)	36.63 (54.69)	33.82 (73.21)	42.22 (119.03)	26.68 (73.05)
AUCg, *M* (*SD*)	2.03 (1.05)	2.03 (1.16)	2.39 (1.22)	2.19 (1.22)	1.92 (0.97)	1.97 (1.14)

Note. Unless stated otherwise, all values are percentages. SMT = stress management training; CAR = cortisol awakening response; AUCg = area under the curve with respect to the ground.
